# The low-molecular-weight heparin, nadroparin, inhibits tumour angiogenesis in a rodent dorsal skinfold chamber model

**DOI:** 10.1038/sj.bjc.6605535

**Published:** 2010-02-02

**Authors:** I Debergh, N Van Damme, P Pattyn, M Peeters, W P Ceelen

**Affiliations:** 1 Department of Surgery, University Hospital, Ghent B-9000, Belgium; 2 Department of Gastroenterology, University Hospital, Ghent B-9000, Belgium; 3 Senior Clinical Investigator of the Research Foundation – Flanders (Belgium) (FWO)

**Keywords:** nadroparin, LMWH, angiogenesis, skinfold chamber, intravital microscopy

## Abstract

**Background:**

Recently, low-molecular-weight heparins (LMWHs) were found to confer a survival advantage in cancer patients. The mechanism underlying this observation is unclear, but may involve inhibition of tumour angiogenesis. We aimed to examine the effects of nadroparin on tumour angiogenesis using a dorsal skinfold window chamber model in the Syrian hamster.

**Methods:**

AMel-3 and HAP-T1 tumours were grown in donor animals and fragments implanted in the window chambers. Animals (*N*=46) were treated with 200 IU of nadroparin or saline for 10 days. Repeated intravital fluorescence microscopy was performed to calculate functional microcirculatory parameters: number (*N*) and length (*L*) of microvessels, vascular area fraction (AF), and red blood cell velocity (*V*). Microvessel density (MVD), fractal dimension, and pericyte coverage were assessed histologically.

**Results:**

Active angiogenesis was observed in control animals, resulting in a significant increase in *N*, *L*, and AF. In nadroparin-treated animals, however, *N* and *L* did not increase whereas AF decreased significantly. Both groups showed an initial increase in *V*, but nadroparin treatment resulted in an earlier decrease in red blood cell velocity over time. Compared with control animals, nadroparin-treated animals showed a significantly lower MVD and fractal dimension but significantly higher pericyte coverage index (PCI).

**Conclusions:**

Taken together, these results suggest that the LMWH nadroparin inhibits tumour angiogenesis and results in microvessel normalisation.

Cancer patients are at risk of venous thromboembolic events (VTEs) induced by the hypercoagulable state associated with malignancy (Mousa, 2004). There is therefore a clear rationale for prophylactic administration of unfractionated heparin (UFH) or low-molecular-weight heparin (LMWH) in these patients (Carrier and Agnes, 2009).

Recent clinical studies have suggested that, independent from its effect on the incidence of VTE, heparin therapy might alter survival in cancer patients (Kakkar *et al*, 2004; Klerk *et al*, 2005).

The potential anticancer mechanisms of LMWH administration remain incompletely understood. Suggested targets include the formation of cancer metastasis, cancer cell adhesion and invasion, immune response, and angiogenesis (Smorenburg and Van Noorden, 2001; Niers *et al*, 2007; Zacharski and Lee, 2008). Angiogenesis is a critical process in survival, growth, and metastasis of a malignant tumour, and is regulated by a number of heparin-binding growth factors such as vascular endothelial growth factor (VEGF) and basic fibroblast growth factor (bFGF). These growth factors bind to heparane sulphate proteoglycans (HSPGs) that are present in both the endothelial cells (ECs) and the extracellular matrix (ECM). Binding of the receptors on the endothelial wall results in proliferation and migration of ECs. Soluble heparins have been shown to compete with angiogenic growth factors for ECM binding sites, and UFH therapy increases the plasma levels of certain growth factors (Folkman *et al*, 1989). In contrast to UFH, LMWHs inhibit binding of heparin-binding growth factors to their endothelial receptors, an effect that depends on the molecule's number of saccharide units (Norrby, 1993; Norrby and Ostergaard, 1996). Fragments of <18 saccharides inhibit the activity of VEGF, whereas fragments smaller than 10 saccharide units reduce bFGF activity (Soker *et al*, 1994). Similarly, in an *in vivo* assay after intraperitoneal VEGF administration, angiogenesis was suppressed by a 5-kDa but not by a 2.5- or 16.4-kDa heparin fraction (Norrby and Ostergaard, 1997).

Most of the experimental data on the anticancer effects of heparins were generated by cell culture experiments. *In vivo* microscopy (IVM) allows longitudinal noninvasive observation of tumour angiogenesis in the living animal. In this study we studied the effects of the LMWH nadroparin on tumour-associated angiogenesis, using a dorsal skinfold window chamber model in the Syrian golden hamster.

## Methods

The experimental protocol was approved by the animal experimental ethical committee of the Ghent University, Ghent, Belgium.

### Animals and tumour model

Male Syrian gold hamsters (Harlan, Horst, The Netherlands) weighing 80–100 g were housed separately in plastic cages with free access to tap water and standard pellet food.

AMel-3 (Fortner's amelanotic hamster melanoma; 50% of the animals) or HaP-T1 (nitrosamine-induced pancreatic cancer in hamsters; 50% of the animals) cancer cell lines were cultured and 1 million cells suspended in 0.1 ml of saline were injected subcutaneously in the proximal hind leg of donor hamsters. When tumours reached a size of 10 mm^3^ (usually after 2–3 weeks), four tumour fragments (±0.5–1 mm^2^) were implanted in the window chamber of acceptor animals at 24 h after dorsal skinfold window chamber implantation.

### Dorsal skinfold window chamber implantation

Hamsters were anaesthetised with intraperitoneal injection of ketamine (Ketalar, Pfizer, Elsene, Belgium) and xylazin (Rompun, Bayer, Diegem, Belgium) and placed on a heating pad. The procedure is described in detail by Endrich *et al* (1980) and Menger *et al* (2002). In brief, a titanium frame is surgically fixed onto a dorsal skinfold of the animal. On one side of the skinfold, a circular area of dermis and subcutis is surgically removed (15 mm diameter) and covered by a circular cover glass. Animal were housed separately and were allowed to recover for 24 h from surgery and anaesthesia before tumour fragment implantation. Window chambers were inspected daily for the presence of air bubbles, inflammation, infection, or vascular thrombosis.

### Experimental therapy

Animals (*N*=23 per group) were treated with daily subcutaneous injections of either 0.07 ml of saline or 200 IU aXa of nadroparin (Fraxiparine, GSK, Genval, Belgium) dissolved in 0.07 ml of saline. Injections started the day before tumour implantation.

### Intravital microscopy

*In vivo* fluorescence microscopy was performed on days 0 (day of tumour implantation), 3, 6, and 9. Unconscious animals (ketamine/xylazin anaesthesia) received an i.v. bolus of 0.1 ml of fluorescein isothiocyanate (FITC)–labelled dextran (20 mg ml^–1^) (Sigma-Aldrich NV, Bornem, Belgium) and were placed on the stage of a modified Olympus BX51WI microscope (Olympus NV, Aartselaar, Belgium). Fluorescence microscopy was performed using an HBO 50W mercury lamp (Osram, Zaventem, Belgium) and a FITC filter set (excitation filter 460–490 nm) for detecting epifluorescent intravascular plasma. Static and dynamic images of the microcirculation were obtained in four different regions in each chamber. Digital images were captured real time on the hard disc of a computer using a high sensitivity digital camera (model C8484-05, Hamamatsu Photonics, Hamamatsu, Japan). Quantitative microcirculatory analysis was performed using a software package (CapImage, H Zeintl Engineering, Heidelberg, Germany). The following parameters were calculated: microvessel length per area (LA; cm cm^–2^), number of microvessels per high-power field (NA; n/HPF × 20), vascular area fraction (AF; %), and microvessel diameter (*D*; *μ*m). In addition, centreline red blood cell velocity (*V*; mm s^–1^) was measured by analysing 10 microvessels per region of interest, randomly chosen among those that crossed a vertical line drawn over the centre of the computer screen, as described by Laschke *et al* (2005). Volumetric blood flow (VQ; pl s^–1^) was calculated from *V* and *D* as VQ=*π* × (*D*/2)^2^ × *V*/*K*, in which *K* (=1.3) represents the Baker–Wayland factor (Baker and Wayland, 1974), and considers the parabolic velocity profile of blood in microvessels. Functional capillary density was not calculated as angiogenic sprouts and buds contain red blood cells without a measurable perfusion and therefore this parameter does not accurately reflect tumour angiogenesis (Torres *et al*, 1995).

On day 9 after implantation of the tumour fragments, animals were killed and the tissue inside the observation chambers was excised for histology. Tissue fragments were fixed in 10% formalin, embedded in paraffin, and 4 *μ*m thick sections were cut and mounted for immunohistochemistry.

### Immunohistochemistry

Factor VIII (von Willebrand factor, FVIII) immunostaining was used to visualise tumour microvessels and calculate microvessel density (MVD), whereas *α*-smooth muscle actin (*α*SMA) was used to identify pericytes and calculate the pericyte coverage index (PCI) as a measure of microvessel maturation.

Serial tissue sections were deparaffinised in xylene, hydrated by serial immersion in ethanol, and subsequently incubated in Proteinase K (Dako, Heverlee, Belgium) for antigen retrieval. Endogenous peroxidase activity was blocked with 3% H_2_O_2_ in methanol. Slides were washed in Tris-buffered saline (TBS)–Tween and treated with UltraSens Block RTU (ImmunoLogic, Duiven, The Netherlands) to inhibit nonspecific antibody binding. Sections were incubated with mouse monoclonal antibodies against FVIII (Dako) or *α*SMA (Abcam, Cambridge, UK) at room temperature for 1 h, rinsed in TBS–Tween, and incubated with secondary antibodies (UltraSens Biotinylated Goat Anti-polyvalent RTU; ImmunoLogic) followed by streptavidin peroxidase (UltraSens Streptavidin Peroxidase RTU; ImmunoLogic) for 10 min each. Visualisation of the immunoprecipitate was performed by adding 3,3′-diaminobenzidine (Biogenex, San Ramon, CA, USA) and counterstaining with haematoxylin. Positive and negative controls were processed simultaneously.

Serial FVIII and *α*SMA-stained sections of dorsal skinfold tissue were entirely scanned for tumour regions (magnification × 20) and digitised. Microvessel density was calculated by digital image analysis using the NIH ImageJ software (version 1.39s, available from http://rsb.info.nih.gov/ij). Using the threshold colour plugin, FVIII- and *α*SMA-positive cells were isolated from background staining and the resulting images converted to binary. Microvessel density was calculated using the ratio of black pixels over the total number of pixels in the binary image, whereas the PCI was calculated as the ratio of *α*SMA-positive pixels *vs* FVIII-positive pixels. The fractal dimension of the microvessel bed was calculated using the ImageJ FracLac plugin. The fractal dimension is a rational number between 1 and 2 (the dimensions of a line and plane, respectively), and has been shown to correlate with the degree of branching, tortuosity, and irregularity of the tumour-associated microvascular network (Dey, 2005).

### Statistical analysis

Data are expressed as mean±s.d. or median (interquartile range). Differences were analysed using Student's *t*-test or Mann–Whitney rank-sum test where appropriate. Results were considered statistically significant when the probability of a type I error was ⩽5%. Statistical analysis was performed with SigmaStat 11.0 (Systat Software, Richmond, CA, USA).

## Results

**Figure 1:**
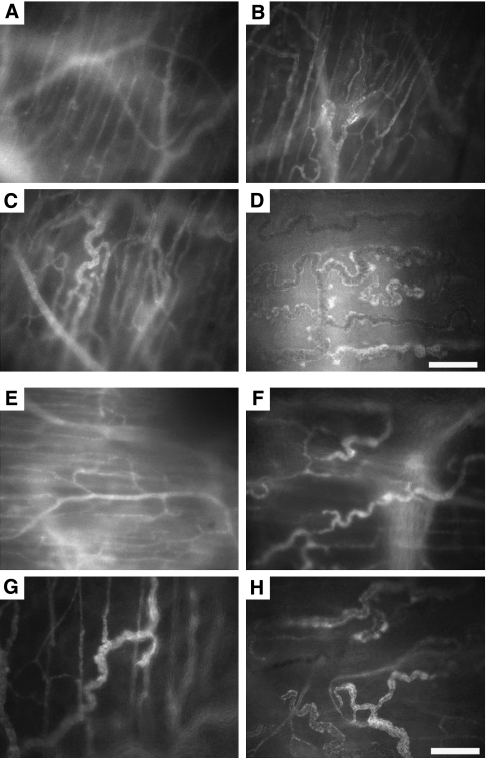
Representative tumour growth and angiogenesis observed for 9 days in a control animal (**A–D**) and a nadroparin-treated animal (**E–H**). The difference in microvessel density is clearly visible, as is the higher number of neovascular buds and sprouts in the control animal (image D). Scale bar=100 *μ*m.

All animals (*N*=46) developed 2–4 macroscopically visible tumours in the observation window, indicating appropriate angiogenesis and viability (Figure 1). As both the dynamic *in vivo* microscopic results and histology parameters with the exception of the PCI did not differ between the HaP-T1 cell line and the AMel-3 cell line (data not shown), statistical analyses were performed on the combined group. Five animals were excluded on the first day of observation because of insufficient optical quality of the window chamber. In all other animals (*N*=41), the skinfold chamber provided excellent image quality and resolution over the duration of the experiment (9 days).

### Effects of nadroparin on tumour-induced angiogenesis

**Figure 2:**
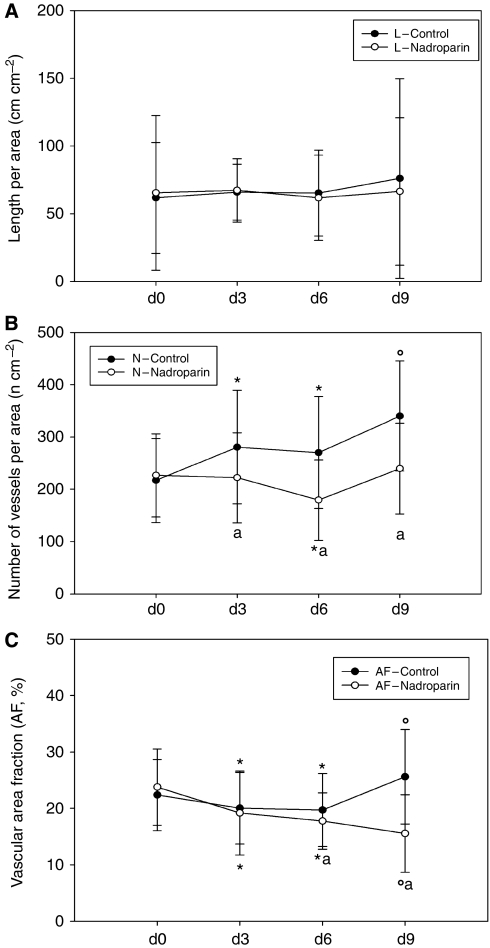
Vessel length (**A**), number of microvessels per area (**B**), and vascular area fraction (**C**) in control and nadroparin-treated animals for 9 days.

The microvascular parameters derived from *in vivo* microscopic observations are summarised in Figure 2 and Table 1. In control animals (*N*=20), the number of microvessels and the vascular area fraction increased significantly whereas a nonsignificant increase in vessel length was observed. In nadroparin-treated animals (*N*=21), however, vessel length and number of microvessels did not change significantly over time whereas a significant decrease in vascular area fraction was noted.

### Effects of nadroparin on the dynamic properties of the tumour vascular bed

**Figure 3:**
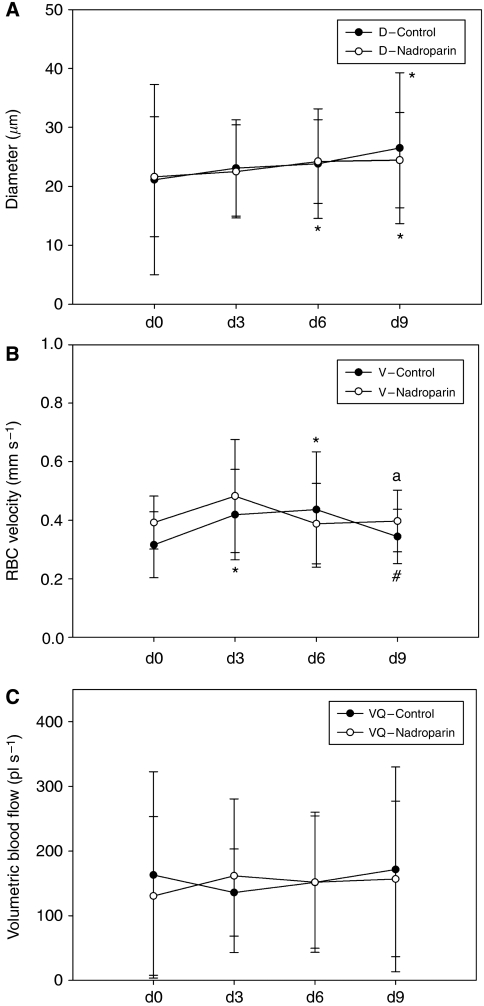
Microvessel diameter (**A**), red blood cell velocity (**B**), and volumetric blood flow (**C**) in control and nadroparin-treated animals for 9 days.

The results of microcirculatory calculations are summarised in Figure 3 and Table 2. In both groups, microvessel diameter increased significantly over time, although in the nadroparin group the increase in diameter between days 6 and 9 was less pronounced. The evolution of microvessel RBC velocity is depicted in Figure 3B. In the control group, velocity increased progressively until day 6, followed by a significant decline at day 9. In the experimental group, however, RBC velocity peaked earlier (day 3) and subsequently stabilised at a lower value. Volumetric blood flow increased significantly over time in control animals, whereas nadroparin-treated animals show an early peak on day 3, followed by stable readings until the end of the experiment.

### Immunohistochemical analysis

**Figure 4:**
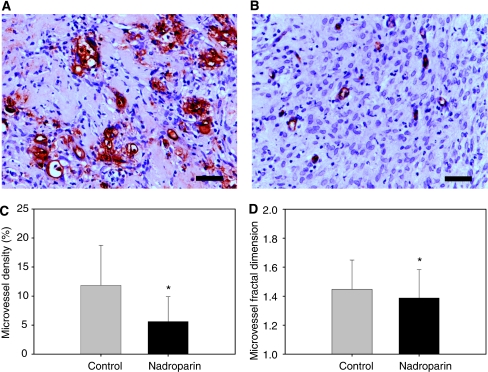
Microvessel density (FVIII staining) and fractal dimension in control and nadroparin-treated animals. (**A, B**) Examples of FVIII-stained microvessels of control and nadroparin-treated animals, respectively. Scale bar=25 *μ*m. (**C**) Microvessel density. Columns= mean; Bars= s.d.; ^*^P<0.001. (**D**) Microvessel fractal dimension. Columns= mean; Bars= s.d.; ^*^P=0.029.

Histological examination of the dissected dorsal skinfolds on day 9 after tumour fragment implantation showed macroscopically vital tumour tissue in all animals. Microvessel quantitative and morphology data are illustrated in Figure 4. The microvessel density (FVIII staining) was 11.1% (6.2–18.1) in the control group and 4.5% (1.8–9.1) in the nadroparin-treated group (*P*<0.001, Mann–Whitney *U*-test). Fractal dimension was significantly higher in control animals than in nadroparin-treated animals (1.5 (1.3–1.6) and 1.4 (1.3–1.5), respectively, *P*=0.029, Mann–Whitney *U*-test).

**Figure 5:**
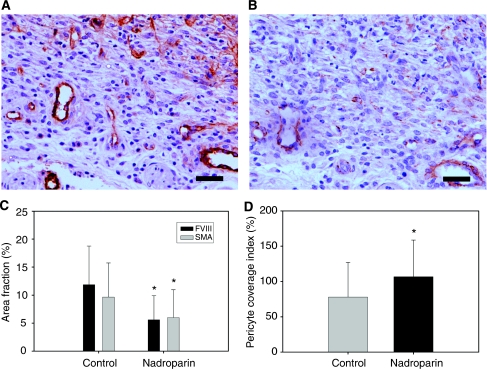
Microvessel maturity (pericyte coverage index) in control and nadroparin-treated animals. (**A, B**) Serial sections of FVIII- and *α*SMA-stained microvessels, respectively, in control hamsters. Weak *α*SMA staining is present around tumour microvessels, illustrating deficient pericyte coverage. Scale bar=25 *μ*m. (**C**) Area fraction (percentage of stained pixels *vs* total number of pixels in binary images) of FVIII- and *α*SMA-stained cells. Columns=mean; Bars=s.d.; ^*^P<0.001. (**D**) Microvessel pericyte coverage index. Columns=mean; Bars=s.d.; ^*^P=0.012.

Data concerning microvessel maturation are depicted in Figure 5. The PCI was 73.6% (39.1–108.5) in untreated animals and 96.8% (74.3–126.1) in nadroparin-treated animals (*P*=0.012, Mann–Whitney *U*-test). The difference in PCI between control and nadroparin-treated animals was more pronounced when the HaPT-1 cell line was used (Table 3).

## Discussion

The results from recent clinical studies in advanced cancer patients have led to increasing interest in the effects of heparins on tumour growth. Klerk *et al* (2005) randomised advanced or metastatic cancer patients to either 6 weeks of nadroparin or placebo, and found a significant overall survival benefit in favour of nadroparin therapy (hazard ratio of mortality 0.75; 95% CI 0.59–0.96).

The underlying mechanisms by which heparins inhibit tumour progression are incompletely understood, and may include inhibition of selectin-mediated cellular adhesion, inhibition of tumour invasion, and induction of cancer cell apoptosis (Smorenburg and Noorden, 2001; Niers *et al*, 2007; Lee, 2007).

One of the causal pathways under scrutiny is the inhibition of tumour-associated angiogenesis. Most *in vivo* angiogenesis assays, such as the mouse cremaster muscle or rat mesenteric window model, allow to observe the microcirculation only once after therapy (Norrby, 2000; Wan *et al*, 2001). The dorsal skinfold chamber model allows noninvasive detailed and repetitive analysis of tumour microvascular properties (Torres *et al*, 1995). Our study is the first to use repetitive observation in a dorsal skinfold window chamber model to assess the effects of an LMWH on tumour-associated angiogenesis in an immunocompetent animal model.

Our results suggest that nadroparin exerts an anti-angiogenic effect *in vivo*, as evidenced by a significantly lower vascular area fraction and MVD when compared with control animals. Moreover, nadroparin-treated tumours showed signs of microvessel normalisation, including a smaller increase in diameter, a higher PCI, and less vessel tortuosity (smaller fractional dimension). Vessel normalisation is a well-characterised early phenotypic effect of anti-angiogenic therapy (Fukumura and Jain, 2008). The difference in vessel diameter and tortuosity may explain the decrease in vascular area fraction over time in nadroparin-treated animals despite the fact that both vessel length and vessel number did not change appreciably over time in this group.

Most of the published studies examining the anti-angiogenic effects of LMWHs have used physiological angiogenesis models such as the rat mesentery or matrigel plug assays. Mousa and Mohamed (2004) showed that the LMWH tinzaparin inhibits angiogenesis in the chick chorioallantoic membrane model by upregulation of the tissue factor pathway inhibitor. Tissue factor, which is the initiator of the extrinsic coagulation pathway, is expressed by many cancer types and was shown to promote tumour angiogenesis through protease-activated receptor 2 (PAR-2) signalling (Belting *et al*, 2004). Similarly, Marchetti *et al* (2008) showed that the LMWHs, enoxaparin and dalteparin, but not unfractionated heparin, significantly inhibited capillary tube formation in a Matrigel assay. Interestingly, the anti-angiogenic potential of LMWHs seems to be depending on the size of the molecule and number of saccharide units. Khorana *et al* (2003) showed that molecules with a weight in the range of 3–6 kDa or >8 saccharide units maximally inhibited angiogenesis in a Matrigel assay. Similar findings were reported by Norrby (2000), who showed that 2.5–5 kDa heparin fragments maximally suppressed VEGF-induced angiogenesis in a rat mesenteric window assay. As the molecular weight of nadroparin is approximately 4.5 kDa, our data confirm the anti-angiogenic potential of heparin fragments within the above-mentioned range.

The basic mechanisms underlying the anti-angiogenic effect of the LMWHs remain to be elucidated. Several potential pathways have been proposed. First, LMWHs suppress TF gene expression and enhance the release of TF pathway inhibitor (Mousa and Mohamed, 2000; Schultz *et al*, 2001). Second, heparin fragments smaller than 18 saccharide units were shown to interfere with the binding of VEGF to its cellular receptor (Soker *et al*, 1994). In addition, heparins inhibit the release of heparanase by malignant tissue (Parish *et al*, 1999, 2001). Heparanase not only creates a pro-angiogenic environment through the release of heparin-bound growth factors (VEGF, bFGF) from the interstitial matrix and upregulation of TF and VEGF expression, but is also a key enzyme in the degradation of this matrix, which renders it ‘permissive’ for neovascular outgrowth (El-Assal *et al*, 2001; Wood *et al*, 2005). Indeed, Collen *et al* (2000) found that structural alterations of the fibrin matrix induced by LMWH reduced the invasion of capillary-forming ECs. Nadroparin may exert a similar effect on ECM properties, as Barner *et al* (1987) found that *in vitro*, LMWH with a mean weight of 4.5 kDa inhibited 99% of heparanase activity.

In addition to a decrease in the number of microvessels, several other structural effects were found to result from nadroparin administration in this study. It is noteworthy that microvascular diameter at day 9 was significantly smaller compared with control animals. Theoretically, this may be explained by the inhibition bFGF and PDFG activity, which both exert a vasodilatory activity (Pukac *et al*, 1997; Takase *et al*, 1999; Millette *et al*, 2005).

The higher PCI and more efficient perfusion that we found in nadroparin-treated animals suggest a normalising effect of this LMWH on the tumour's vascular bed. As a consequence, there may be a role for LMWH to enhance drug delivery of concurrently administered cytotoxic therapy. In patients with pancreatic cancer and small cell lung cancer, administration of LMWH enhanced the efficacy of combined chemotherapy (Icli *et al*, 2002; Altinbas *et al*, 2004).

Several limitations apply to the interpretation of the current results. First, although it may be assumed that inhibition of tumour angiogenesis in this model translates into inhibition of tumour growth, this could not be assessed because of the short time frame used (9 days). Second, implantation of window chambers invariably induces a certain degree of inflammation that might confound the findings related to tumour angiogenesis. Finally, it is unclear to what extent findings in a heterotopic animal tumour model will translate into relevant clinical effects in patients.

In addition to the uncertain basic mechanisms by which heparins interfere with tumour angiogenesis, several other questions remain open. It is unclear whether the anti-angiogenic effects of LMWHs depend only on their molecular weight and number of saccharide units, or also on other physicochemical properties such as manufacturing process and degree of sulphation. In addition, although most of the mechanisms shown thus far suggest a rather generic mode of action, preclinical studies suggest that the antitumour effects of heparins depend on the cancer cell type (Niers *et al*, 2009). Finally, the LMWH dose and duration in the clinical setting remain to be determined.

In conclusion, nadroparin inhibits tumour-associated angiogenesis and normalises microvessel structure in this immunocompetent tumour model using the dorsal skinfold chamber. Further study is required to determine whether direct effects on EC proliferation and modelling, changes in the structure of the extracellular matrix, or both explain our observations.

## Figures and Tables

**Table 1:** Evolution of microvessel length, number, and area fraction over time

	**Day 0**	**Day 3**	**Day 6**	**Day 9**
*Control (N=20)*				
L (cm cm^–2^)	61.8±41	65.9±21	65.2±32	76.0±74
N (*n* cm^–2^)	216.8±80	280.8±108^*^	270.3±107^*^	340.2±105^**^
VAF (%)	22.4±6.3	20±6.4^*^	19.7±6.5^*^	25.6±8.4^**^
				
*Nadroparin (N=21)*				
L (cm cm^–2^)	65.4±57	67.2±23	61.8±31	66.5±54
N (*n* cm^–2^)	226.8±80	222.2±86^***^	179.4±77^*,***^	238.9±87^***^
VAF (%)	23.8±6.7	19.2±7.4^*^	17.8±5.0^*,***^	15.6±6.9^**,***^

Abbreviations: L=microvessel length; N=number of microvessels; VAF=vascular area fraction. ^*^P<0.05 *vs* day 0. ^**^P<0.05 *vs* day 0, 3 and 6. ^***^P<0.05 *vs* untreated animals at corresponding time points. Data represent mean±s.d.

**Table 2:** Evolution of microvessel diameter, red blood cell velocity, and volumetric blood flow over time

	**Day 0**	**Day 3**	**Day 6**	**Day 9**
*Control (N=20)*				
D (*μ*m)	21.2±16.1	23.1±8.1	23.8±9.3	26.5±12.8^*^
V (mm s^–1^)	0.3±0.1	0.4±0.1^*^	0.4±0.2^*^	0.3±0.1^**^
VQ (pl s^–1^)	163.1±159	135.8±67^*^	151.6±108^*^	171.5±158^*^
				
*Nadroparin (N=21)*				
D (*μ*m)	21.6±10.1	22.5±7.9	24.2±7.1^*^	24.4±8.0^*^
V (mm s^–1^)	0.4±0.1^***^	0.5±0.2	0.4±0.1	0.4±0.1^***^
VQ (pl s^–1^)	130.4±122^***^	161.7±118	151.9±102	156.8±120^*^

Abbreviations: D=microvessel diameter; V=red blood cell velocity; VQ=volumetric blood flow; Data represent mean±s.d. ^*^P<0.05 *vs* day 0. ^**^P<0.05 *vs* days 3 and 6. ^***^P<0.05 *vs* untreated animals at corresponding time points.

**Table 3:** Immunohistochemistry results

**Group**	**Control (*N*=20)**	**Nadroparin (*N*=21)**	***P*-value**
*AMel-3*			
FVIII	9.4 (5.2–15.1)	3.8 (1.4–6.6)	<0.001
SMA	5.1 (3.8–8.0)	1.7 (1.4–7.9)	0.020
PCI (%)	95.7 (54.5–117.9)	111.8 (63.3–134.3)	NS
			
*HaP-T1*			
FVIII	11.7 (6.9–19.9)	5.8 (2.4–10.7)	<0.001
SMA	10.9 (7.4–17.2)	5.8 (2.6–10.4)	<0.001
PCI (%)	76.5 (39.7–111.7)	108.3 (80.2–157.8)	0.003

Abbreviations: FVIII=factor VIII staining; SMA=smooth muscle antigen staining; PCI=pericyte coverage index. Data represent median (interquartile range).
